# Open Acromioclavicular Repair With a Suture Cerclage Tensioning System: A Case Series

**DOI:** 10.7759/cureus.34018

**Published:** 2023-01-20

**Authors:** Alvarho J Guzman, Shane Rayos Del Sol, Therese Dela Rueda, Sarah Jenkins, Caleb Shin, Ryan Haratian, Patrick McGahan, James Chen

**Affiliations:** 1 Orthopedic Surgery, Advanced Orthopedics & Sports Medicine, San Francisco, USA

**Keywords:** orthopedic surgery, case-series, orthopedic sports medicine, ac joint, shoulder injuries

## Abstract

Introduction

Numerous surgical techniques to address a type III and type V acromioclavicular (AC) joint separation have been described in the literature, but a preferred standard approach is still in debate. Current approaches include anatomic reduction, coracoclavicular (CC) ligament reconstruction, and anatomic reconstruction of the joint. In this case series, subjects received a surgical approach that avoids metal anchors and utilizes a suture cerclage tensioning system to achieve adequate reduction.

Surgical technique

An AC joint repair was achieved with a suture cerclage tensioning system, which allows the surgeon to apply a specific amount of force on the clavicle to achieve adequate reduction. This technique repairs the AC and CC ligaments, restoring the anatomy of the AC joint while avoiding some of the common risks and disadvantages associated with metal anchors.

Methods

From June 2019 to August 2022, 16 patients underwent repair of the AC joint with a suture cerclage tension system. Inclusion criteria included the diagnosis of type III or type V AC joint separation with another concomitant injury, acute and chronic injury, and patients who attended all their postoperative visits. Exclusion criteria included patients who lost to follow-up or patients who missed any of their postoperative visits. Radiographic images were taken during each subject’s preoperative and postoperative visits, and the CC distance was measured to determine the integrity of the all-suture cerclage repair.

Results

Of the 16 patients included in this case series, radiographic images taken during each subject’s postoperative visit showed a stable construct with little changes in the CC distance. The average change in CC distance when comparing the two-week and one-month postoperative follow-up is 0.2mm. The average change in CC distance when comparing the two-week and two-month postoperative follow-up is 1.45mm. The average change in CC distance when comparing the two-week and four-month postoperative follow-up is 2.6mm.

Conclusion

Overall, an AC joint repair with the suture cerclage tension system can be a viable, cost-effective technique for restoring vertical and horizontal stability. Although follow-up, larger-scale studies are required to determine the biomechanical integrity of the construct with an all-suture approach, this case series presents 16 subjects whose postoperative radiographic images showed only a small change in CC distance at two to four months after surgery.

## Introduction

The acromioclavicular (AC) joint is a diarthrodial joint that articulates between the lateral end of the clavicle and the acromion of the scapula forming the medial portion of the anterior shoulder [1,2]. As a plane-type synovial joint, the AC joint primarily allows gliding movement but allows an additional degree of axial rotation and anteroposterior movement as it attaches the scapula to the thorax [3]. AC joint injuries mainly occur as a direct blow to the lateral acromion while the arm is abducted or after a fall onto the ipsilateral extremity in an adducted or outstretched position [1-4]. AC joint injuries account for approximately 9% of all shoulder girdle injuries and most commonly occur from high-impact contact sports such as hockey, wrestling, and rugby [1]. Moreover, AC joint injuries comprise an estimated incidence of1.8 per 100 per year, with men between 20 and 39 years being 8.5 times more likely than women to suffer injury [5]. Controversy over the management of type III AC joint separations exists without clear consensus and the decision to proceed operatively vs. nonoperatively is determined on a case-by-case basis from individual patient circumstances [1,2,4,6]. Depending on the type of AC joint separation, injury to the joint can result in the rupture of the AC ligament or coracoclavicular (CC) ligaments, which control horizontal and vertical stability, respectively. The surgical technique utilized in this case series is a variation of previous anatomical reconstruction techniques in the literature. It aims to re-establish the natural anatomy of the AC joint with a suture wrapped beneath the coracoid and through the clavicle at the approximate locations of the CC ligaments while also restoring horizontal stability.

Due to the complexity of AC joint dislocations and the insufficient evidence behind its optimal operative method, the goal of this case series is to evaluate the functional outcomes and success of a suture cerclage tensioning system to provide an anatomic repair of type III and V AC joint separation [7].

To our knowledge, no published studies or case series have evaluated the efficacy of operative treatment for type III and V AC joint separations utilizing a suture cerclage tensioning system surgical technique. This case series will investigate the functional outcomes of an anatomic AC joint repair using a suture cerclage tensioning system for type III and type V AC joint separations and establish the reliability and effectiveness of this surgical technique in AC joint repair literature through radiographic evaluation.

## Materials and methods

Surgical technique

**Figure 1:**
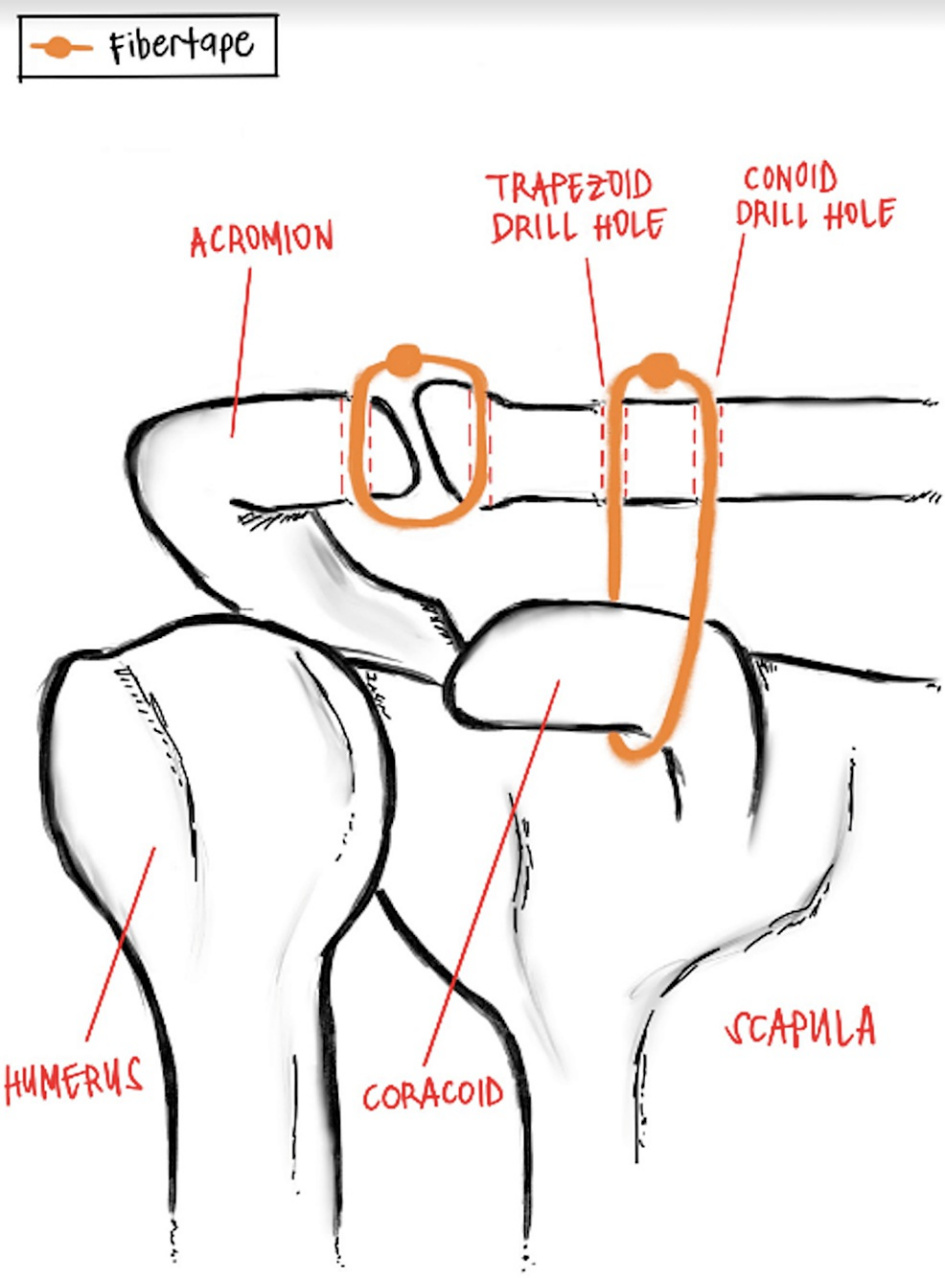
Labeled depiction of open AC joint repair with suture cerclage tensioning system. Image credit: Therese Dela Rueda

Youn et al. describe the AC joint repair with a Fibertape cerclage tensioning system in detail with surgical photos and an accompanying video [7]. The patient is positioned in a beach chair position, and the head and all bony prominences are supported and well padded. The surgical field is prepped and draped in the usual sterile fashion. An incision extending over the AC joint is created and the surgeon carefully dissects down through the deltopectoral fascia to the superior border of the clavicle for proper visualization of the distal clavicle and AC joint separation. Blunt dissection is performed until the superior aspect of the coracoid can be felt. A graft passing instrument (GPI) (Arthrex, Naples, FL) is loaded with a nitinol wire, and the instrument is hooked around the coracoid. The nitinol wire is fed through the GPI and is passed beneath the coracoid, and the nitinol wire is used to shuttle #2 Fiberwire under the coracoid. Once the attachments of the conoid and trapezoid CC ligaments are determined, a drill is used to create two 3.0mm bicortical drill holes through the clavicle at the conoid and trapezoid ligament attachments. A nitinol wire is used to shuttle each tail of the #2 fiberwire through the conoid and trapezoid drill hole. Using the #2 Fiberwire as a shuttling suture, Fibertape from the cerclage system (Arthrex) and a #5 Fiberwire are shuttled through the conoid drill hole, under the coracoid, and through the trapezoid drill hole. The Fibertape is loaded and fed through the Fiberlink suture of the cerclage system, creating a self-locking mechanism that allows the sutures to irreversibly slide when applying tension. 70 lbs of tension were used until the AC joint was visually reduced. The Fibertape is then loaded onto a Fibertape cerclage tensioner (Arthrex, Naples, FL), which allows the surgeon to remove any slack in the construct and control the amount of reduction. Each tail is positioned anterior and posterior to the clavicle, and the #5 Fiberwire is tied down over the clavicle to back up the self-locking knot created by the Fibertape cerclage. Reduction is confirmed visually with intraoperative radiographic images once the acromion and clavicle are lined up anatomically and compared to the contralateral side. Figure 1 shows the AC repair with the suture cerclage system.

This case series review was approved by the Institutional Review Board. This review is a single-centered study that enrolled patients from a private-practice system located in San Francisco, CA. Patients were enrolled from June 2019 to August 2022. Enrollment of patients was done consecutively during the enrollment period, and they were included in the study if they satisfied the inclusion criteria. Relevant dates for data collection included preoperative and postoperative visits in the clinic at two weeks (PO1), one month (PO2), two months (PO3), and four months (PO4). Patients enrolled in the case series included those with the diagnosis of type 3 or type 5 AC joint injury who underwent repair with the suture cerclage tensioning system. No type 4 injures were included since we did not see any patients with this injury given its rarity in clinical practice. In addition, patients who presented with a concomitant injury such as a distal clavicle fracture associated with the initial injury were included. Exclusion criteria included patients lost to follow-up or patients who did not attend at least three of their postoperative visits. An open AC joint repair surgery with a suture cerclage tension system was provided to all enrolled patients. If the patient’s initial injury was greater than six months, the injury was classified as chronic, and the surgery involved the addition of an allograft. In all of these cases, the Fibertape (Arthrex) is a substitute for a torn CC ligament. The allograft is also a cerclage but is used as a supplement for the Fiber Tape (Arthrex). Moreover, if a distal clavicle fracture was present, it was addressed with internal fixation during the surgery. These procedures were completed by a board-certified fellowship-trained sports medicine orthopedic surgeon in an outpatient surgery center. Postoperative instructions were provided to each patient. From 0-6 weeks postoperatively, patients were instructed to wear a postoperative sling at all times and to engage in pendulum swings in the affected extremity. At six weeks postoperatively, the patient was instructed to initiate formal physical therapy with a focus on a range of motion and to progress to strengthening exercises as tolerated. Preoperative and postoperative anterior-posterior (AP) x-ray images of the affected shoulder for each patient were collected at the relevant postoperative dates. Standard AP radiographs were used to measure the CC distance. The degree of AC joint separation was determined by measuring the CC distance in each subject’s preoperative radiographic images. Furthermore, the integrity of the AC joint repair was measured by obtaining the CC distance during each subject’s postoperative visit. The CC distance is defined as the distance between the superior cortex of the coracoid to the inferior border of the clavicle. The CC distance of each radiographic image was measured by two reviewers and confirmed by the surgeon.

## Results

Over the enrollment period, 16 patients were diagnosed with a type III or type V AC joint injury and underwent an AC joint repair with the suture cerclage tensioning system, resulting in adequate reduction. Two patients were excluded due to failure to attend a minimum of three postoperative visits. Of the 14 patients included in the study, five patients were diagnosed with a type III AC joint separation and nine patients were diagnosed with a Type V AC joint separation. The average CC distance measured at two weeks postoperatively was 8.2mm. At one month postoperatively, the average CC distance measured was 8.8mm. At two months postoperatively, the average CC distance measured was 9.7mm. Of the 16 enrolled subjects, 9 patients were present during their four-month postoperative visit, and the average CC distance measured was 11.02mm. When comparing the average CC distance during postoperative visits, the average change in CC distance between the two weeks and one month postoperative visit was +0.2mm. The average change in CC distance between two weeks and two months postoperative visit was +1.45mm. Furthermore, among the four patients who attended the four months postoperative visit, the average change in CC distance between two weeks and four months postoperative visit was 2.6mm.

**Figure 2:**
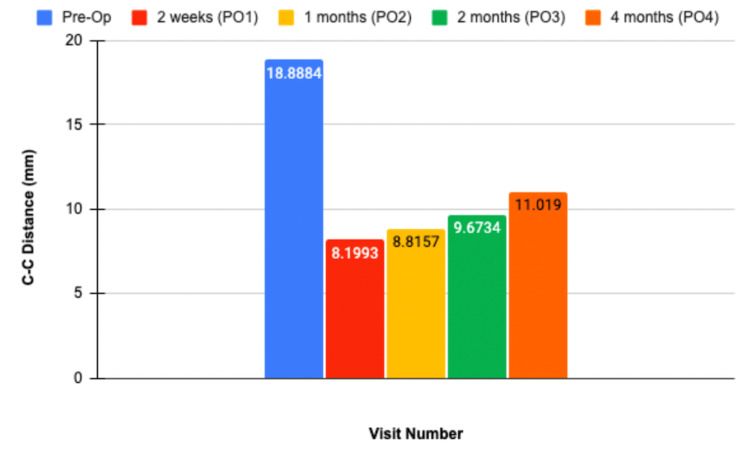
Average CC distance measured among all enrolled patients. The average CC distance measured at two weeks, one month, two months, and four months postoperatively is 8.2mm, 8.8mm, 9.7mm, and 11.02mm, respectively. Image credit: Alvarho Guzman

**Figure 3:**
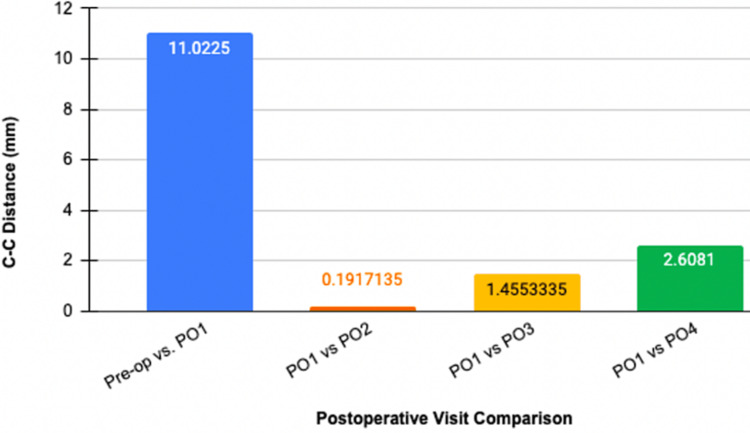
Average CC distance change of all patients over the postoperative course in comparison to first postoperative visit (PO1) two weeks following surgery. Image credit: Alvarho Guzman

**Figure 4:**
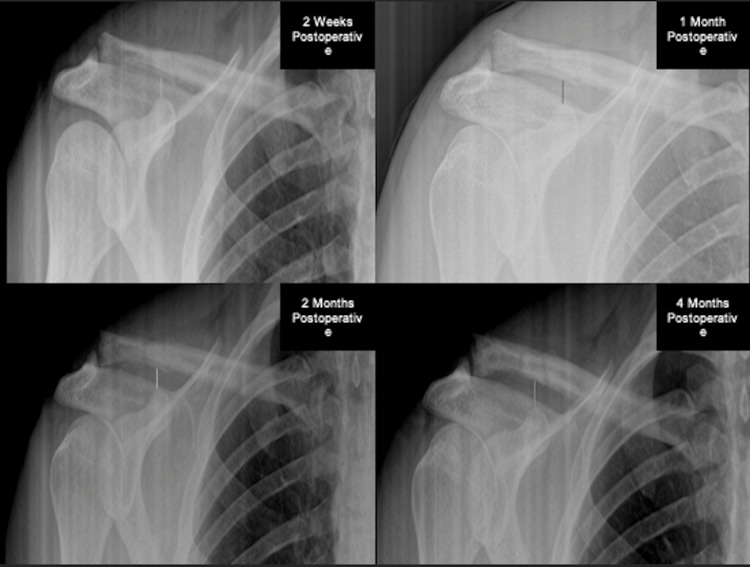
AP radiographs of subject 3 with two weeks, one month, two months, and four months postoperative radiographs available. At the subject’s two weeks visit, the CC distance is 7.8mm. During the one-month visit, the CC distance is 8.0mm. At the two months visit, CC distance is 8.1mm. At the four months visit, the CC distance slightly increased to 10.3mm. Image credit: Alvarho Guzman

Complete patient information including age, sex, physical exam, diagnosis, surgical procedure, and CC distance measured during the preoperative and postoperative visits are included in Table 1. Changes in individual CC distance seen in subsequent postoperative visits compared to two weeks postoperatively are seen in Table 2. The average CC distance measured at two weeks, one month, two months, and four months in all subjects can be seen in Figure 2. The average CC distance change of all patients over the postoperative course in comparison to the PO1 two weeks following surgery is depicted in Figure 3, which displays how much reduction was achieved from surgery and any superior migration of the clavicle following repair. Radiographic images of subjects before and after AC joint repair can be seen in Figure 4.

**Table 1:** Information containing patient history and mechanism of injury, physical exam, diagnosis, surgical procedure, and coracoclavicular distance measured during patients’ preoperative and postoperative visits. Image credit: Alvarho Guzman

Patient #	Patient Information	Physical Exam	Diagnosis	Procedure	Coracoclavicular distance
1	53 yo male. Two weeks history of left shoulder injury after falling off a bike	Tenderness over the AC joint. Pain with active and passive ROM.	Type III AC joint separation with distal clavicle fracture	AC Joint Repair with suture cerclage system and distal clavicle open reduction internal fixation	Pre-op: 16mm 2 weeks PO: 2mm 1 month PO: 2.8mm 2 months PO: 5.0mm
2	38 yo male. 1 week history of right shoulder pain due to fall while skiing	Tenting with ecchymosis and tenderness to palpation over the AC joint. ROM deferred due to pain.	Type V AC joint separation	AC Joint Repair with suture cerclage system	Pre-op: 19.8mm 2 weeks PO: 1.7mm 1 month PO: 1.6mm 2 months PO: 1.8mm
3	34 yo male. 2 weeks history of right shoulder pain due to a fall while snowboarding	Obvious deformity and tenderness to palpation over AC joint. Pain with passive ROM. Positive Neer’s	Type V AC joint separation	AC Joint Repair with suture cerclage system	2 weeks PO: 7.8mm 1 month: 8.0mm 2 months PO: 8.1mm 4 months PO: 10.34mm
4	50 yo female. 10 months history of right shoulder pain after falling off a horse. Pain worsened after weightlifting 6 months after initial injury	Deformity over AC joint without ecchymosis. Full active ROM with pain associated with forward flexion and external rotation. No rotator cuff weakness	Type III AC joint separation	AC Joint Repair with suture cerclage system with allograft	Pre-op: 7.5mm 2 months PO: 6.9mm 1 month PO: 6.8mm 2 months PO: 7.0mm 4 months PO: 7.6mm
5	36 yo male. 2 months history of left shoulder pain following a fall off a bicycle. Reports loss of consciousness following the fall. 6 weeks of physical therapy with limited improvement	Obvious deformity and tenderness to palpation over AC joint. Positive vertical instability	Type III AC joint separation	AC Joint Repair with suture cerclage system	Pre-op: 20.2mm 2 weeks PO: 9.3mm 1 month PO: 12.2mm 2 months PO: 13.0mm 4 months PO: 13.8mm
6	26 yo male. 1 week history of right shoulder pain after falling off a scooter	Obvious deformity and tenderness to palpation over AC joint. Full active ROM with pain	Type III AC joint separation	AC Joint Repair with suture cerclage system	2 weeks PO: 8.4mm 1 month PO: 9.4mm 2 months PO: 10.8mm 4 months PO: 13.6mm
7	32 yo male. 1 day history of right shoulder pain after falling off a bike	Ecchymosis and tenderness to palpation over the AC joint. ROM limited due to pain	Type V AC joint separation	AC Joint Repair with suture cerclage system	Pre-op: 22.4 mm 2 weeks PO: 4.6mm 1 month PO: 6.5mm 2 months PO: 12.0mm
8	25 yo male. 6 day history of left shoulder pain after sustaining direct impaction fall while snowboarding.	Palpable defect along the AC joint. Abndormal contour of left shoulder relative to the right.	Type V AC joint separation.	AC Joint Repair with suture cerclage system	Pre-Op: 17mm 2 weeks PO: 9.37mm 1 month PO: 9.86mm 2 month PO: 10.06mm 4 month PO: 10.39mm
9	31 yo male. One day history of left anterior shoulder pain after sustaining a fall on the left upper extremity.	Obvious deformity , swelling, and tenderness to palpation over AC joint and distal clavicle.	Type 5 AC joint separation.	AC Joint Repair with suture cerclage system	Pre-Op: 20.93mm 2 weeks PO: 11.36mm 1 month PO: 11.44mm 2 month PO: 11.98mm 4 month PO: 12.11mm
10	28 yo male. 3 day history of left shoulder pain after sustaining fall while snowboarding.	Tenderness to palpation over AC joint and acromion process. Obvious deformity notable.	Type III AC joint separation.	AC Joint Repair with suture cerclage system	Pre-Op: 20.18mm 2 weeks PO: 8.91mm 1 month PO: 9.11mm 2 month PO: 9.21mm 4 month PO: 9.33mm
11	44 yo male. 3 week history of right shoulder pain after fall off of truck at work.	Tenderness to palpation over AC joint and distal clavicle. Visible elevation of distal clavicle relative to contralateral side.	Type V AC joint separation.	AC Joint Repair with suture cerclage system	Pre-Op: 18mm 2 weeks PO: 11.28mm 1 month PO: 11.51mm 2 month PO: 11.77mm 4 month PO: 12.07mm
12	28 yo male. 1 week history of left shoulder pain after direct impaction fall while snowboarding.	Tenderness to palpation over AC joint. Positive cross body abduction test.	Type V AC joint separation.	AC Joint Repair with suture cerclage system	Pre-Op: 22.17mm 2 weeks PO: 9.35mm 1 month PO: 9.86mm 2 month PO: 9.96mm 4 month PO: 10.03mm
13	27 yo male. 2 day history of left shoulder pain after being thrown to the ground during judo competition.	Tenderness to palpation over AC joint. Positive anterior apprehension test.	Type III AC joint separation.	AC Joint Repair with suture cerclage system	Pre-Op: 21.76mm 2 weeks PO: 12.28mm 1 month PO: 12.59mm 2 month PO: 12.92mm
14	23 yo male. 2.5 month history of right shoulder pain after snowboarding injury.	No tenderness to palpation over AC joint. 2cm superior displacement of right AC joint relative to contralateral side.	Type V AC joint separation.	AC Joint Repair with suture cerclage system	Pre-Op: 24mm 2 weeks PO 11.98mm: 1 month PO: 12.15mm 2 month PO: 12.27mm

**Table 2:** Individual CC distance at the 2 weeks, 1 month, and 2 months postoperative visits and changes in CC distance seen during a patient’s postoperative course compared to the initial 2 week postoperative visit.

Patient #	CC distance at 2 Weeks PO	CC distance at 1 month PO	Change compared to 2 weeks	CC distance at 2 months PO	Change compared to 2 weeks	CC distance at 4 months PO	Change compared to 2 weeks
1	2mm	2.8mm	+0.8mm	5.0mm	+2.2mm	-	-
2	1.7mm	1.6mm	-0.1mm	1.8mm	+0.1mm	-	-
3	7.8mm	8.0mm	+0.2mm	8.1mm	+0.3mm	10.3mm	+2.3mm
4	6.9mm	6.8mm	-0.1mm	7.0mm	+0.1mm	7.6mm	+0.8mm
5	9.3mm	12.2mm	+2.9mm	13.0mm	+0.8mm	13.8mm	+1.6mm
6	8.4mm	9.4mm	+1mm	10.8mm	+1.4mm	13.6mm	+5.2mm
7	4.6mm	6.4mm	+1.8	12.0mm	+5.6mm	-	-
8	9.37mm	9.86mm	+0.49mm	10.06mm	+0.69mm	10.39mm	+1.02mm
9	11.36mm	11.44mm	+0.08mm	11.98mm	+0.62mm	12.11mm	+0.75mm
10	8.91mm	9.11mm	+0.2mm	9.21mm	+0.3mm	9.33mm	+0.42mm
11	11.28mm	11.51mm	+0.23mm	11.77mm	+0.49mm	12.07mm	+0.79mm
12	9.35mm	9.86mm	+0.51mm	9.96mm	+0.61mm	10.03mm	+0.68mm
13	12.28mm	12.59mm	+0.31mm	12.92mm	+0.64mm		
14	11.98mm	12.15mm	+0.17mm	12.27mm	+0.29mm		

 

## Discussion

In 1984, Rockwood et al. developed a radiographic classification system (I-VI) for AC joint injuries based on the direction and degree of displacement relative to the contralateral shoulder [8]. Type I and II AC joint separations are treated nonoperatively with rest and shoulder immobilization. Type IV, V, and VI AC joint separations are characterized by significant displacement and require operative treatment to restore the anatomical position and normal shoulder function [1,2,4]. While there are numerous techniques used to treat AC joint separations, a consensus standard surgical approach is yet to be reported [2,9]. Current approaches for reconstructing the AC joint include anatomic reduction, CC ligament reconstruction, and anatomical reconstruction, with each approach having its own set of advantages and challenges [10]. Although anatomic reduction and CC ligament reconstruction both successfully establish vertical stability, a significant disadvantage to both approaches is continued instability in the horizontal plane [11]. Furthermore, anatomic reduction requires two stages with implantation and removal of the hardware, and approximately 20% of patients fail to maintain adequate reduction after one year [12]. Although CC ligament reconstruction is a single procedure that reduces the risk of infection and neurovascular injury, the current literature in biomechanical outcome studies is lacking [10]. Anatomic reconstruction is the only approach that restores horizontal and vertical stability. Although additional biomechanical studies are also needed to evaluate the integrity of this approach, early studies comparing an anatomic reconstruction versus an isolated CC ligament reconstruction show improved outcomes with the former [7,13,14].

The Fibertape cerclage system provides a robust repair of the AC joint which is carefully achieved through the use of the cerclage tensioner and by verification of anatomic reduction through fluoroscopic imaging [7]. As opposed to the well-known hook-plate stabilization technique which achieves a reduction in the vertical plane but requires subsequent surgery to remove the hardware, this technique utilizes two sturdy sutures under the coracoid to achieve anatomic reduction and does not require follow-up surgery. The function of the CC ligaments is restored with this technique while avoiding associated risks of migration. We achieve this result by passing suture through the clavicle drill holes and underneath the coracoid [7]. The clavicle cerclage and tensioning system described in this case series demonstrated satisfactory results in our cohort of patients. The technique used in this report showed similar if not slightly superior results compared to other techniques described in the literature such as Li et al., which reported a CC distance average of 11.2mm+/-1.8mm at the final follow-up [15]. The follow-up duration in the Li et al. study was a mean +/- SD follow-up of 39.3 +/- 8.9 months (range, 24.7-36.3 months) while our follow-up period was 3.29 months, highlighting a limitation in our study being its short follow-up duration. The clavicle cerclage technique described in this manuscript largely eliminates the need for donor allograft except in cases of chronic AC joint injury and creates a lasting AC joint reduction while reducing cost and minimizing complications such as the need for revision surgery due to loss of reduction. Furthermore, subjects included in this case series show only a slight increase in CC distance following their AC joint repair. As a whole, the AC joint repair proved to be a viable construct that maintained CC distance. At the one-month and two-month postoperative visits, the average CC distance measured was 8.8mm and 9.7mm, respectively. Although the four months postoperative visit shows an increase in CC distance, this average only includes nine of the 16 enrolled subjects. Overall, some superior migration of the clavicle is expected as the repair heals and as the patient initiates formal physical therapy.

Many different techniques have been described to repair an AC joint separation including CC screw, hook plate, button fixation, and ligament reconstruction [16,17]. A recent review by Phadke et al. highlighted the potential advantages and disadvantages of the different techniques [17]. The hook plate was found in several studies to cause shoulder impingement and can lead to an increased risk of complications due to the need to remove the plate after healing [17,18]. A recent systematic review looking at case studies published on the efficacy of coracoacromial ligament transfer for the repair of AC joint dislocations found that there is a high rate of construct failure [19]. The Arthrex Tightrope system remains a viable technique, but there has still been a high rate of complication and construction failure reported, including fracture of the coracoid and instability in the horizontal plane [20]. Additionally, anytime metal implants are used there is an increased chance of bony erosion or irritation, a potential complication that is avoided with the suture cerclage construct presented in this case series.

Tomlinson et al. present a similar technique to ours using allograft instead of suture to loop around the clavicle recreating the CC ligaments and demonstrating favorable results [21]. Ours differs in that it allows the surgeon to precisely control the amount of tension applied in reducing the AC joint during the repair, does not require an allograft, and was performed open rather than arthroscopically. While our technique is slightly more invasive, repairing the AC joint without an allograft cuts down on surgical costs and potential complications associated with allograft use. Additionally, an open approach allows the surgeon to apply a specific amount of force on the clavicle to achieve adequate reduction. Furthermore, one randomized control trial by Lee et al. found no significant difference in AC joint reconstruction surgery outcomes when allograft was used to when it was not, further supporting our decision to use a suture-based cerclage rather than using a graft [22].

While clavicle cerclage has been utilized in the past as a portion of an AC joint reconstruction, it has rarely been used as the primary fixation of the reduction while using only suture for the cerclage fixation [21,23]. For example, a retrospective study by Sandmann et al. found open AC Joint Reconstruction using AC and CC PDS provides satisfactory clinical results in the majority of cases [24]. The repair described in this paper uses #2 Fiberwire rather than PDS and uses the Arthrex tensioning system to control the reduction [23,25]. Similar to previous studies which used PDS for primary fixation of reduction, our technique showed good clinical outcomes at each sequential postoperative visit with no reported re-dislocations or re-injuries.

One limitation of our study is the small sample size (16 patients) and the short-term nature of our follow-up (mean follow-up duration of 3.29 months). Since we did continue to see some lengthening of the AC distance in follow-up x-rays, a follow-up study is required to determine how much the CC distance continues to change while the patient continues to gain function and strength in the shoulder. However, it is important to note that none of these patients reported a decrease in functionality due to this finding. In order to best assess the quality of this technique in the future, further follow-up of these patients is required. Another limitation of our study is that our cases were a mix of acute and chronic AC joint separation. In the future, it would be interesting to separate acute and chronic injuries and analyze them separately to assess if our construct works better in a subset of cases, although one study by Ladermann et al. found that there was no significant difference in a variety of patient-reported outcomes in patients treated within two weeks of injury or greater than two weeks after injury [26].

## Conclusions

Overall, there is a lack of quality research to advocate for a standard surgical repair of the AC joint. Our paper seeks to describe the outcomes of our unique AC joint repair technique in a small cohort of patients. Larger scale studies with longer postoperative follow-ups will be required before recommendations can be made for one surgical technique. We conclude that our clavicle cerclage technique with the Arthrex tensioning system produces favorable results in a small cohort of patients and is a viable option for the repair of Rockwood type III and type V AC joint dislocations.
